# Validation of a Novel Wearable Multistream Data Acquisition and Analysis System for Ergonomic Studies

**DOI:** 10.3390/s21248167

**Published:** 2021-12-07

**Authors:** Luca Ascari, Anna Marchenkova, Andrea Bellotti, Stefano Lai, Lucia Moro, Konstantin Koshmak, Alice Mantoan, Michele Barsotti, Raffaello Brondi, Giovanni Avveduto, Davide Sechi, Alberto Compagno, Pietro Avanzini, Jonas Ambeck-Madsen, Giovanni Vecchiato

**Affiliations:** 1 Henesis s.r.l., 43123 Parma, Italy; a.bellotti@camlintechnologies.com (A.B.); s.lai@camlintechnologies.com (S.L.); a.mantoan@camlintechnologies.com (A.M.); m.barsotti@camlintechnologies.com (M.B.); g.avveduto@camlintechnologies.com (G.A.); d.sechi@camlintechnologies.com (D.S.); a.compagno@camlin.tech (A.C.); 2 Camlin Italy s.r.l., 43123 Parma, Italy; l.moro@outlook.it (L.M.); k.koshmak@camlintechnologies.com (K.K.); rbrondi@gmail.com (R.B.); 3 Institute of Neuroscience, National Research Council of Italy, 43125 Parma, Italy; marchenkova.anna@gmail.com (A.M.); pietro.avanzini@gmail.com (P.A.); 4 Toyota Motor Europe, 1114 Bruxelles, Belgium; jonas.ambeck@toyota-europe.com

**Keywords:** wearable device, ergonomics, EEG, bio-potentials, behavior

## Abstract

Nowadays, the growing interest in gathering physiological data and human behavior in everyday life scenarios is paralleled by an increase in wireless devices recording brain and body signals. However, the technical issues that characterize these solutions often limit the full brain-related assessments in real-life scenarios. Here we introduce the Biohub platform, a hardware/software (HW/SW) integrated wearable system for multistream synchronized acquisitions. This system consists of off-the-shelf hardware and state-of-art open-source software components, which are highly integrated into a high-tech low-cost solution, complete, yet easy to use outside conventional labs. It flexibly cooperates with several devices, regardless of the manufacturer, and overcomes the possibly limited resources of recording devices. The Biohub was validated through the characterization of the quality of (i) multistream synchronization, (ii) in-lab electroencephalographic (EEG) recordings compared with a medical-grade high-density device, and (iii) a Brain-Computer-Interface (BCI) in a real driving condition. Results show that this system can reliably acquire multiple data streams with high time accuracy and record standard quality EEG signals, becoming a valid device to be used for advanced ergonomics studies such as driving, telerehabilitation, and occupational safety.

## 1. Introduction

The development of mobile devices for the acquisition of brain and body signals [1,2], as well as of Brain Computer Interfaces (BCI) [3,4,5,6] for ecologic applications, such as driving or neuro-rehabilitation, requires HW/SW flexible platforms able to reliably acquire, synchronize and record multiple streams of different signals, compact enough not to limit movements or interactions, easy to be operated and interfaced with off-the-shelf sensors, and cost-effective [7].

The growing development of BCI solutions has been paralleled by a flourishing increase of devices allowing to record brain and body signals wirelessly, i.e., in out-of-the-lab conditions. BCI research has usually focused on clinical applications to facilitate disabled individuals to interact with the environment. Typical examples in this field encompass the design of brain-controlled intelligent wheelchair with the capability of automatic navigation [8], non-invasive framework to achieve the neural control of a robotic device for continuous target tracking [9], applications to restore motor functions by inducing activity-dependent brain plasticity for post-stroke individuals [10] (see [11] for a review). In addition, BCI-based solutions are gaining attention for developing applications supporting industrial performance by optimizing the cognitive load of working operators, facilitating human-robot interactions, and making operations in critical conditions more secure [4]. However, these solutions often involve technical issues that limit the full exploitation of BCI and, more generally, brain-related assessments in real-life scenarios. A multistream recording is not ubiquitous among commercial, wireless recording devices, and the number of available solutions decreases even more when searching for the collection of multimodal signals (e.g., the acquisition of bio-potentials together with the position of body joints or videos from cameras). In addition, the most informative bio-potential is the electroencephalographic signal (EEG) that continues to be the most difficult to record in a real-life environment, as it reflects an uncontrolled mixture of several neural processes [12] (contrarily to what happens in lab settings) that might be hard to dissociate. Further, EEG signal is often contaminated by different types of noise whose weight typically increases moving from the lab to real-life settings [13].

Another fundamental aspect of these platforms is the capability to flexibly cooperate with the largest number of devices, regardless of the manufacturer, and overcome the possibly limited resources of recording devices. Given the richness of the recordable datasets, a fundamental aspect is to ensure a precise and stable temporal synchronization among sources, which is a key factor for determining the inter-dependencies among signals. Even if open-source solutions have been developed for this purpose, such as the Lab Streaming Layer (LSL, see https://github.com/sccn/labstreaminglayer, accessed on 29 November 2021) envisioned platforms should include redundant communication protocols and solutions to include all the recording devices within the synchronization chain, such as those based on wireless channels not overseen by LSL. Finally, most of the available commercial solutions take care of the recording and online analysis stages with no possibility to control the delivery of experimental stimulations to the subject. While this kind of procedure may seem limited to lab-like experimental settings, it is worth considering that well-established neural features can also be monitored and exploited “into the wild”, returning valuable information about the subject’s mental and physical state.

Starting from these premises, here we introduce the Biohub platform, a hardware/software (HW/SW) integrated wearable system for multistream synchronized acquisitions. We validated this platform in three stages, comprising a characterization of the multistream synchronization, an electrophysiological validation of its EEG recordings through a comparison with a medical-grade high-density device, and the realization of a Brain-Computer-Interface (BCI) as a use case in a real driving condition. Specifically, the multistream synchronization is achieved by complementing state-of-the-art solutions such as LSL with an easy to perform calibration step. The electrophysiological validation consists of the in-lab recording and comparison of EEG features assessing the reactivity of occipital and motor rhythms, and event-related potentials. Finally, we demonstrate the usability of the Biohub in ecological settings with a BCI experiment performed in a real driving scenario.

Due to the complexity of the underlying brain processes and the technological perspectives possibly impacting the market, driving is one of the most studied scenarios targeted by the field of neuroergonomics [14,15]. Several systems have been developed to decode brain activity anticipating salient actions during driving, such as braking [16,17] and steering [18,19,20]. Then, it is still critical to measure the brain and body activity of the driver, assess his/her mental state to prevent workload, fatigue and drowsiness [21], and decode neurophysiological temporal dynamics associated with movement preparation and execution. These features would eventually permit the tracking of the driver behavior in real scenarios and complement the information coming “from the outside”.

In the present study, we will show how the novelty of the Biohub platform mainly resides in its capability to embed, within the same device, several qualities. First, its open architecture facilitates the integration of virtually any sensors, safeguarding the richness of the acquired datasets. Indeed, the Biohub can simultaneously and synchronously record a broad set of signals comprising biopotentials such as electromyography (EMG), electrocardiography (ECG), and EEG, behavioral signals through eye-tracking devices, and other signals that can be collected by a smartphone, such as GPS, data coming from inertial sensors and video streams. Second, the Biohub can also be used as a programmable interface for stimuli presentation, with the additional possibility to control the stimuli delivery based on contextual signals. Finally, the Biohub is suitable for running online data analyses, which are to date more and more fundamental in moving ergonomic studies out of the classical laboratory conditions, i.e., in more ecological and thus demanding real-life scenarios (e.g., driving).

## 2. Materials

### 2.1. The Platform

The platform (Biohub in the following) is an HW/SW acquisition platform specifically designed and implemented to record bio-potentials and other signals through a synchronised multistream architecture. Biohub has been designed to be fully modular, supporting a variable number of nodes, input sources, and devices. Although streams can be heterogeneous in data format and production rate, the inter modules’ data synchronization is guaranteed through the LSL protocol, which sends data over local networks based on TCP protocol. The central node is a data collector module in charge of recording, synchronizing, and processing the streams coming from different modules and devices, which can run on a PC or on a Linux-based embedded system. For these purposes, the Jetson TX2 module (NVidia, Santa Clara, CA, USA) has been specifically selected to design a compact, wearable, and portable solution.

### 2.2. SW Architecture of the Biohub Platform

The architecture consists of several nodes, network-connected, in charge of streaming data coming from different devices using LSL. LSL (https://labstreaminglayer.readthedocs.io/, accessed on 29 November 2021) is a collection of tools that includes a transport layer library, a recording program, file importers, and programs that allow data from a variety of sources to be shared over a local network. It is a general-purpose and cross-platform library that manages the transparent streaming and synchronization of multiple streams originating from different devices connected to the same local network. In particular, time synchronization relies on two pieces of data that are being collected alongside the actual sample data:

1. A timestamp for each sample that is read from a local high-resolution clock of the origin device (in most cases, the resolution is better than a microsecond);
2. Out-of-band clock synchronisation information transmitted, with each data stream, to the receiving computer. It consists of the momentary clock offset between the two devices estimated at periodic intervals using an NTP-like algorithm.

The timestamps of the different streams can be remapped onto a shared time-domain (that of the receiving machine) based on the history of clock offsets. A running timestamp correction factor is computed for each stream, fitting a linear model on the history of clock offsets. This correction is then applied to remap the raw timestamps onto the shared time dimension.

Synchronized data are recorded using LabRecorder, an LSL tool that resolves all the reachable LSL streams on the networks and records them on an XDF file. Other nodes have been developed in order to collect data from a variety of sources, specifically: (1) an Android application to collect data from Bluetooth IMUs, record videos, GPS location; (2) a PC application to record data from different EEG devices and a gaze tracker (Pupil Labs).

LSL provides accurate online synchronization across nodes. However, the achievement of sub-ms data alignment requires to measure and compensate for the latencies that originated outside the control of the LSL protocol, including those caused by wireless communication protocols, either proprietary or non-TCP/IP or non-LSL WiFi (not LSL managed network transmissions), to cite cases contemplated in Biohub. If measured, these offsets can be embedded into the LSL header, in order for the receivers to compensate for such offsets, thus reaching perfect alignment. A method for such estimation was developed and is described below.

An Orchestrator application has been developed to minimize the experimenters’ effort to launch all the required nodes on multiple devices and handle a session recording. This component can remotely launch all the nodes specified in a configuration file, ensure that all streams are correctly received, and start data session acquisition.

**Figure 1:**
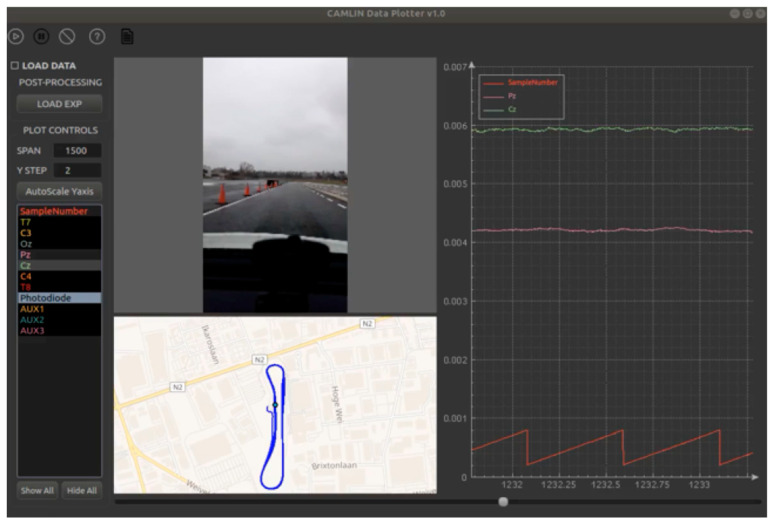
Multimodal GUI. A screenshot of the GUI while using the Biohub in a car experiment is shown. On the left, the user can use the menu to start the recording, load pre-recorded datasets, or control plot parameters and data sources. The central panel shows the synchronized video captured from the smartphone camera pointing at the road and the route reconstructed by the GPS module of the Android smartphone. On the right, two EEG signals and one control signal are plotted according to the selection carried out by the user.

A Multimodal GUI has been also developed, to visualize all the LSL streams across the network. Numeric streams are plotted in a common central plot, while text streams are visualized as timestamped single events. Visualization plugins have been developed for some stream types-like EEG, ECG, EMG, quaternions, videos-to display data in a custom way; for instance, quaternions are visualized as floating cubes, GPS locations are displayed on a map, whereas electrophysiological streams have custom plot display modes. The Multimodal GUI can handle both online streams in real-time, as well as previously acquired XDF recordings. Figure 1 shows the data from an in-car experiment acquisition.

### 2.3. Supported Devices in the Biohub Platform

#### 2.3.1. Jetson TX2

In our portable configuration, the central node of the architecture of the Biohub platform is hosted on a Jetson TX2 module (i.e., host-pc), mounted on an Orbitty carrier board (Connecttech, Guelph, Canada), where all data are collected, recorded and, if required, locally processed. LSL’s LabRecorder runs on this node, receiving via WiFi all the LSL streams over the LAN. The Jetson is also embedded with an inertial measurement unit (IMU), which streams inertial data via LSL up to 1 kHz data rate and characterizes the initial alignments. The NVidia GPU hosted onboard allows to run online data analyses.

In our mobile configuration, we developed specific Linux drivers to allow the Jetson and Orbitty boards to recognize all the devices supported by the Biohub. A battery pack ensures power in mobility, an LTE USB modem ensures Internet connectivity, and a belt allows to wear the whole system. The resulting compact solution is portable into a vehicle and even wearable for “in mobility” applications.

The main benefit of having the NVidia GPU hosted onboard is that Biohub can be used both in the acquisition and analysis phase of ergonomic studies in real scenarios due to its capability of data recording and online signal processing.

#### 2.3.2. Android Smartphone

Modern smartphones are equipped with different sensors such as GPS, IMUs, cameras, proximity, and light sensors providing information on the user. An Android application was developed to allow the different modules to stream data using the LSL protocol in the Biohub platform. A Samsung Galaxy S8 (Samsung, Suwon, South Korea) was used in our setup. However, different smartphones have been tested with consistent performance. We also integrated with LSL the following smartphone modules:

- Embedded GPS module, which can stream location data at 2 Hz via LSL;
- Embedded Cameras, whose 30 fps-compressed videos are saved locally on the device, while the frame number is streamed via LSL. Capture parameters are configurable;
- Embedded IMU module, which streams inertial data at 500 Hz via LSL. Raw data from accelerometers, magnetometers and gyroscopes as well as fused data (e.g., quaternions and Euler angles) are supported. Moreover, an LSL support was developed to send the data from additional IMUs (Metawear-R, MBientlab, San Francisco, CA, USA). These IMUs were connected via Bluetooth Low Energy (BLE) to the smartphone, with a configurable (25–100 Hz) rate depending on the number of connected IMUs (from 1 to 5) sharing BLE bandwidth. These additional IMU sensors allow the driver to have their arms and legs monitored during the driving.

#### 2.3.3. OpenBCI

A Cyton board (OpenBCI, Brooklyn, NY, USA) was selected as a bio-potential (EEG, EMG, or ECG) acquisition board due to its good performance compared to its cost. The Cyton board was connected to a WiFi shield (OpenBCI, Brooklyn, NY, USA) to allow for the data streaming at higher rates (up to 1 kHz) compared to the default BLE connection allowing for a maximum streaming frequency of 250 Hz. The Cyton can manage the streaming of 8 (1 kHz) or 16 channels (500 Hz) and the data streaming from an embedded IMU sensor via LSL. Moreover, we developed a dedicated software that uses the Brainflow library (https://brainflow.org/, accessed on 29 November 2021) to manage the Cyton board configuration on the Biohub platform. Although we configured the Brainflow library for the Cyton board, integrating this library in our system allows Biohub to be ready for the support of different board types.

### 2.4. EEG Headset Plugins of the Biohub Platform

The Biohub platform was configured to support both commercial and custom EEG headsets. Among commercial systems, we natively support the Enobio 20 (Neuroelectrics, Barcelona, Spain) and g.Nautilus research EEG device (g.tec medical engineering, Schiedlberg, Austria); the former streams 20 EEG channels over BLE, while the latter streams 32 EEG channles over proprietary WiFi, both require an external PC to generate and transmit LSL streams over the local network at 500 Hz, packeted with signals from the embedded IMU sensor.

The Biohub was also combined with a new headset, specifically designed for EEG acquisition in a vehicle. The headset is composed of an elastic EEG cap with EEG electrodes placed in correspondence of 7 recording sites (Fpz, Fz, Cz, Pz, Oz, C3, C4) of the head (earlobes used as reference and ground in this configuration). Dry electrodes from Neuroelectrics were used for the acquisition and held on the corresponding sites by a net of adjustable elastic bands, providing an ergonomic head fit and adequate pressure to ensure a good adherence with the skin, and at the same time being comfortable enough to guarantee the tolerability of long recordings (their impedance with the skin remained below 300 kΩ for the entire recording duration). Electrodes were connected through low-impedance cables to a Cyton OpenBCI board, which streams them at 500 Hz.

**Figure 2:**
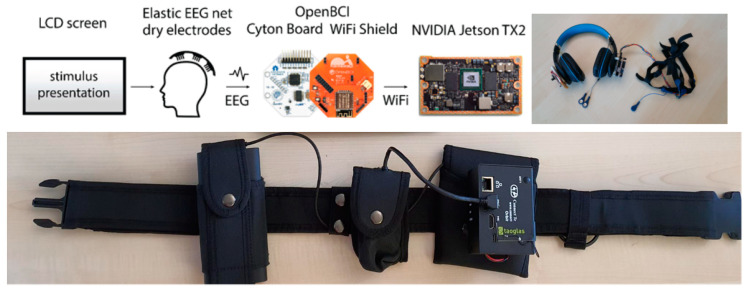
Top: pipeline of signals within the Biohub architecture (left); EEG headset plugin (right). Bottom: mobile configuration of the Biohub (from left to right: power bank, USB modem, embedded system based on Jetson TX2 and Orbitty carrier board in a metallic case).

A headphone (Beexcellent Q2 wireless headset) is used as the headset frame: the OpenBCI with its wifi-shield is screwed on the left side, whereas a recharging LiPo battery is placed on the right side of the headphone. The EEG cables run inside the headphone through a dedicated area specifically designed to reduce cables burden and improve wearing comfort. Figure 2 shows the Biohub platform (a schematic view on the left, the EEG headset plugin hardware implementation on the right).

## 3. Methods

In the following sections, we present the procedures adopted to (i) characterize the multistream synchronization of Biohub, (ii) electrophysiologically validate the EEG headset in a standard laboratory setup, (iii) set up a BCI for a use case in a real driving scenario.

### 3.1. Characterisation of Multistream Synchronisation

The online streams synchronisation is based on the widely adopted and already validated LSL middleware. As such, the missing aspect is the estimation of latencies of: (1) the internally developed nodes and plugins (Android LSL for Bluetooth IMUs, client for Cyton OpenBCI, client for g-Tec) as they necessarily rely on wireless transmission not managed by LSL, respectively, non-TCP/IP, non-LSL WiFi, WiFi proprietary; (2) the BLE channel between the external IMUS and the smartphone.

The simple procedure we describe relies on the fact that all the physical devices in Biohub contain an internal inertial unit. Applying a mechanical perturbation to all of them physically bounded to the same rigid structure and a correlation-based analysis allows assessing and modeling the offset among individual devices. Testing accelerometers is enough given that all other streams are packeted alongside timestamped accelerometric data before streaming over LSL.

Data streams synchronization performance of the Biohub was thus validated through the recording and analysis of accelerometers’ streams originating from 4 devices (which are the collectors of many more data streams) and 4 external IMUs:

- Android smartphone (mobile, Samsung Galaxy S8), (streamed at 500 Hz)
- OpenBCI, Cyton board (streamed at 500 Hz)
- g.Nautilus research EEG cap (streamed at 500 Hz)
- Jetson TX2 (internal accelerometer sampled at 1 kHz)
- External IMUs (Metawear, Streaming at 25 Hz to the Smartphone)

Synchronization tests were performed along four sessions each time starting from a cold restart of all devices involved. During an acquisition session, each device streamed both the accelerometer signal and the other signals, ranging from bio-potentials for Cython and g.Nautilus (e.g., EMG and EEG) to the video frame number, for a total of 25 different streams.

During the synchronization tests, the devices were rigidly mounted along the perimeter of a circular panel (diameter = 500 mm, thickness = 3 mm) placed above a wood bench, and mechanical perturbations were provided. The mechanical perturbations consisted random movements and strokes at the center of the panel (inner circle with diameter = 25 mm). We adopted both modalities to assess the usability of a random movement (highest ease for the final user) but with the de-risking presence of an easily re-alignable impulsive signal.

Accelerometers’ data coming from the devices were loaded from the recorded files using the pyxdf module (version 1.13, see https://github.com/xdf-modules/pyxdf, accessed on 29 November 2021) with default parameters (enabled clock synchronization and jitter removal). Then, data were resampled to 1000 Hz, and a cross-correlation analysis was performed on the data chunks where a perturbation was present. This procedure returned statistics of the offsets among streams after each system reset.

### 3.2. Electrophysiological Validation of EEG Helmet

#### 3.2.1. Participants

Twenty-one participants (9 males, 28.71 ± 4.14 years old) were recruited for the electrophysiological validation of the EEG recordings performed with the Biohub system. All participants had a normal or corrected-to-normal vision and did not report any neurological or psychiatric diseases at the time of the EEG recordings. They signed a written informed consent to participate in the study. Approval for the study was obtained from the local Ethical Committee (“Comitato etico Unico per la provincia di Parma”).

#### 3.2.2. Experimental Setup and Tasks

The Biohub electrophysiological validation procedure consisted in the administration of four tasks to test the reliability of the system to measure the reactivity of (i) the occipital alpha and (ii) sensorimotor rhythms, (iii) the steady-state visual evoked potential (SSVEP), and (iv) the P300 event-related potential (ERP). Each participant completed an EEG recording session (around 25 min) with both EGI and Biohub systems on the same day with a short break between sessions (around 5–10 min). The EGI and Biohub recordings were counterbalanced and performed inside a silent cabin which guarantees a noise reduction ≥25 dB for frequencies ≥125 Hz, as well as a magnetic shielding of ≥30 dB and electrical shielding ≥50 dB for frequencies ≥100 kHz. Experimental tasks were implemented using the Psychopy 2.0 and E-Prime 2.0 software, for the Biohub and EGI recordings, respectively. A standard 24-inch LCD computer monitor with 1920 × 1080 resolution and 60 Hz refresh rate was used to administer the tasks. Participants were seated 1 m from the monitor during the two recording sessions. Each experimental session started with a 1-min free blinking period subsequently used for blink identification and removal. Participants were free to initiate each task at his/her own pace. Each participant was given a full written description of all tasks and performed a 5-min training before starting. Participants’ behavior was monitored during the experiment to ensure that they accomplished the tasks correctly.

Eyes open-closed task. Participants were asked to close their eyes after hearing a tone and keep them closed until the tone repeats, then keep the eyes open and look at the fixation cross in the center of the screen. The duration of the eyes’ open-closed periods was equal to 5 s. This trial was repeated 10 times.

Motor task. Participants were instructed to perform hand clutching movements with both hands simultaneously while the word “move” was shown on the screen (4 s) and rest while the word “rest” was shown. The rest period had a random duration uniformly distributed between 8 and 12 s to avoid learning the time course of the task and prevent movement anticipation. This trial was repeated 25 times.

SSVEP task. SSVEPs were elicited using two visual pattern reversal stimulations through a square checkerboard pattern modulated at 5 and 6 Hz to produce the primary SSVEP responses at the alternation rate of the checkerboard pattern, which is twice the modulation frequency (i.e., 10 and 12 Hz, respectively) [22,23,24,25]. It was previously shown that the checkerboard pattern produces a more pronounced SSVEP response compared to a regular flicker stimulus modulated at the same frequency [25]. These stimulation frequencies were chosen as the more frequently used for SSVEP-based BCI systems [24] and to accommodate the monitor’s 60 Hz refresh rate without entering the 15–25 Hz range considered the most important seizure-provocative [26]. There were three runs of 10 s for each stimulation frequency, with a 7 s rest period between the trials.

**Figure 3:**
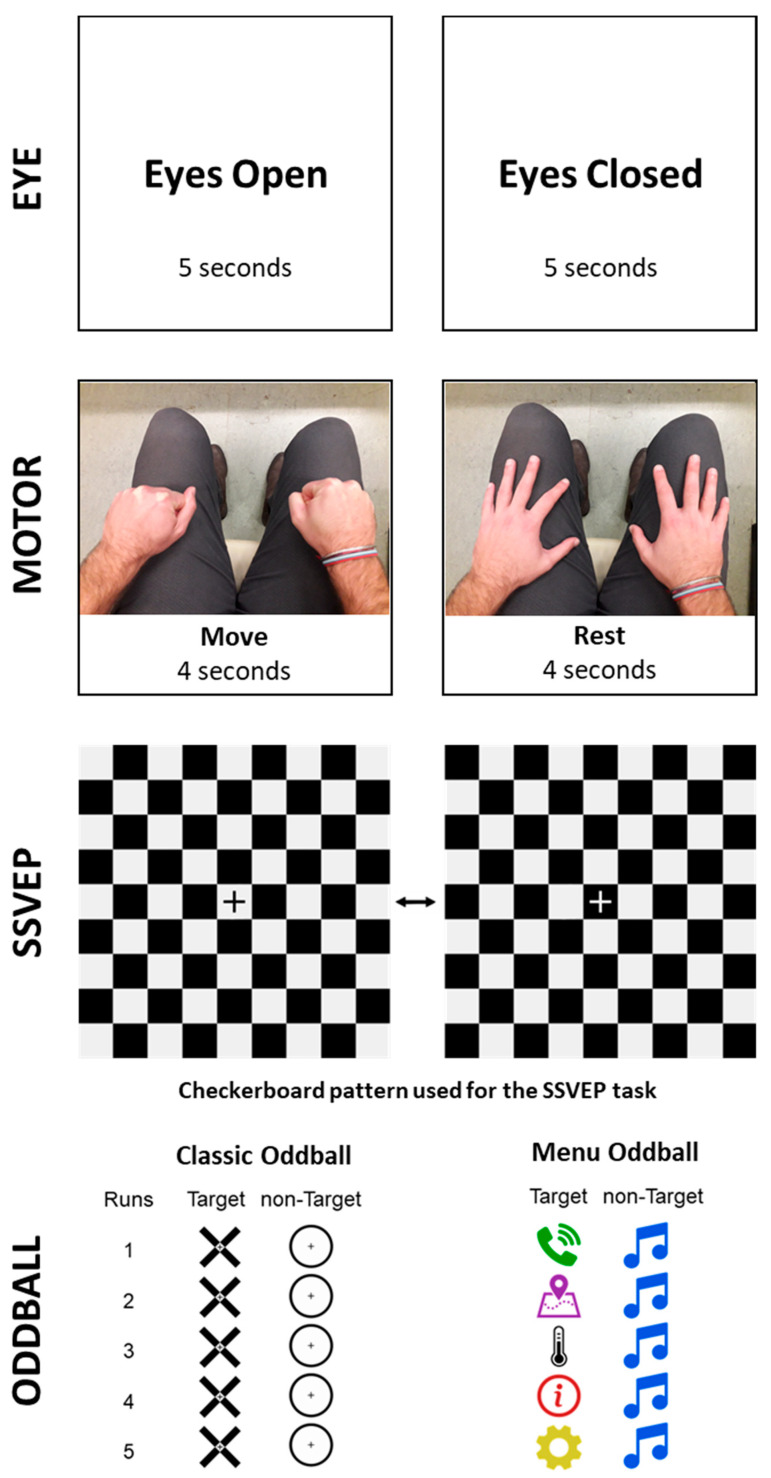
Schematic representation of the Eye and Motor tasks. Stimuli used in the SSVEP and Oddball tasks.

Oddball tasks. Two versions of a two-class visual oddball paradigm were implemented to elicit and analyze the P300 ERP [27]. In both versions, stimuli were presented one by one in the center of the screen for 200 ms with a subsequent 400 ms interval before the next stimulus [28]. The target/non-target ratio was set to 1/5, and each run had 10–12 target appearances (randomly chosen) and a corresponding (60–72) number of non-target presentations. Each version of the task consisted of 5 runs with a resting period in between runs. Participants were instructed to focus on and mentally count the target stimuli and ignore the non-target ones. In the first oddball task (‘classic’ oddball), target and non-target stimuli were a black cross and a black circle on a gray background, respectively. These stimuli remained constant for all runs, sessions and subjects. In the second oddball task, we used a set of 6 different icons from a hypothetical car infotainment menu as stimuli (‘menu’ oddball). For each participant, each icon was randomly assigned to one out of six colors–red, blue, green, yellow, magenta or black–without repetitions. From this set of stimuli, one was set as a non-target for all the runs of a session, and the other five icons were used as targets, one for each of the five runs. Therefore, the target stimuli changed at each run, while the non-target stimuli remained the same for each participant across the acquisition sessions with the EGI and Biohub systems. Figure 3 provides a visual description of the four tasks.

#### 3.2.3. EEG Recordings

EGI system. Continuous EEG was acquired using the 128-channel Geodesic EEG System (Electrical Geodesics, Inc., Eugene, OR, USA) and the HydroCel Geodesic Sensor Net, which arrays 19 electrode sensors (AgCl-coated electrodes) in a geodesic pattern over the surface of the head at the equivalent 10–20 system locations. Consistent positioning was achieved by aligning the Sensor Net with skull landmarks (nasion, vertex, and pre-auricular points). Low-noise EEG data were obtained using high-input impedance amplifiers (Net Amps300) with sensor-skin impedances maintained below 50 kΩ. The signal was digitized at a sampling rate of 500 Hz (0.01 Hz high-pass filter) and recorded with a vertex reference, the impedance of which was kept below 10 kΩ. EEG data were stored in raw format in NetStation.

Biohub system. Continuous EEG was acquired using the Biohub system and sampling the scalp at seven recording sites (Fpz, Fz, Cz, Pz, Oz, C3, C4) having the corresponding channels on the HydroCel Geodesic Sensor Net. Raw EEG signals were collected at 500 Hz with OpenBCI, the left earlobe was used as a reference, and the dry electrodes’ impedances were kept below 300 kΩ. Signals were wirelessly transmitted to the Jetson, which created LSL streams with EEG data, markers, and information about the lost packets and the temporary loss of connection. The streams were saved as XDF files using LabRecorder.

#### 3.2.4. EEG Analysis

EEG data of both EGI and Biohub recording sessions were imported into the Python environment and subjected to the following analysis using the MNE Python library. Due to the wireless data transmission, during the Biohub recordings, data loss occurred in some trials. These were automatically rejected from the analysis if more than 20 consecutive samples were lost (4.2 ± 3.8% trials discarded due to data loss). EEG signals were referenced to the common average reference and band-pass filtered between 1 and 30 Hz. Blinks correction was performed using the Fpz channel for blinks identification and implementing a signal-space projection (SSP) technique [7].

Eyes open-closed task. EEG epochs were extracted around the stimulus onset using the [0.5, 5] s window for both eyes open and closed conditions. The first 0.5 s from the stimulus onset were not included in the analysis to account for the subject’s response time. The EEG artifacts rejection was performed in two steps through a semi-automatic procedure. First, epochs were thresholded based on the signal amplitude to remove those with unlikely high/low values outside the [−50, 50] μV range. Then, the power spectral density (PSD) was calculated for each epoch in the range of [1, 30] Hz window using the PSD multitaper function (Python MNE library [29,30]). Epochs with PSD > 60 dB in the [20, 30] Hz window were rejected. In addition, each epoch was fitted with a linear fit model and rejected if the r squared linear correlation coefficient was larger than 0.85. Finally, the trials automatically selected for rejection were visually inspected and validated manually. Differences between the two acquisition systems were tested by comparing the PSD values for each frequency bin within the alpha band in the range of [8, 12] Hz with the *t*-test.

Motor task. EEG epochs were extracted using the [0, 4] s window for the clutching condition and [2, 6] s of the resting period. The EEG artifacts rejection was performed in two steps through a visually inspected semi-automatic procedure. The EEG artifacts rejection was performed with the two steps semi-automatic procedure described in the Eye task analysis paragraph. Then, we computed event-related spectral perturbation (ERSP; event-related shifts in the power spectrum) [31]. Time-frequency decomposition was determined using Morlet wavelets with linearly increasing frequencies from 1 to 30 Hz. The number of wavelet cycles also increased from 1 to 30 in linear steps. Then, dB conversion is performed, using the pre-event interval of [−6, 0] s. Motor activity and baseline were compared using dependent sample t-statistics and nonparametric permutation testing, corrected for multiple comparisons by cluster mass correction with randomization of 1000 and a statistical threshold of 0.05 [32].

SSVEP task. Three overlapping (50%) 5 s long epochs were extracted from each 10 s trial for both 5 Hz and 6 Hz conditions. The EEG artifacts rejection was performed in two steps through a visually inspected semi-automatic procedure. The EEG artifacts rejection was performed with the two steps semi-automatic procedure described in the Eye task analysis paragraph. The PSD was calculated using Welch’s method (Python SciPy package; Hanning window, 2000 samples segments, 1000 sample overlap, and 5000 points Fast Fourier Transform). The results were then averaged between subjects to compute the grand averages for both acquisition setups.

Oddball tasks. EEG epochs were extracted around the stimulus onset using the [−0.2, 0.7] s window. The EEG artifacts rejection was performed in two steps through a visually inspected semi-automatic procedure. The [−0.2, 0] s window was used for baseline correction. Grand-average of target and non-target EEG trials were computed for the EGI and Biohub systems, and the P300 ERP was compared for each time bin within the time window [250, 550] ms using the *t*-test.

To compare the statistical power of the EEG features between the EGI and Biohub systems as a function of the number of trials, we used a permutation procedure in which we varied the number of trials contributing to the EEG feature. In this case, 1000 permutations were randomly selected for each number of trials to compute the statistical significance using the *t*-test. For this analysis, we used the peak amplitude of the PSD in the [8, 12] Hz frequency window at the Oz electrode for the eyes open-closed task; the peak amplitude of the PSD in the [8, 12] Hz frequency window at the C3 and C4 electrodes for the motor task; the peak amplitude of the PSD at 10 and 12 Hz at the Oz electrode for the two SSVEP tasks; the P300 peak amplitude in the time window [250, 550] ms at the Cz channel for the two oddball tasks.

All *t*-tests were performed with the significance threshold set at 0.05 and were Bonferroni corrected for multiple comparisons.

### 3.3. BCI Application

#### 3.3.1. Participants

Ten participants (9 males and 1 female, age 37.8 ± 7.42) were selected for the experiment presented in [33]. All participants had normal or corrected-to-normal vision, did not report any neurological or psychiatric diseases at the time of the recordings, and signed a written informed consent to participate in the study.

#### 3.3.2. Experimental Setup and Tasks

The experiment aimed to validate the Biohub platform in driving conditions and consisted in the implementation and operation of a prototype BCI allowing to control the infotainment menu of a car exploiting the ERPs elicited by an implementation of the oddball paradigm. The menu was composed of 6 icons, and they were presented on a small display one at a time. The icon selection was performed by paying attention to the carousel and identifying the appearance of the desired (deviant) one to elicit a P300.

**Figure 4:**
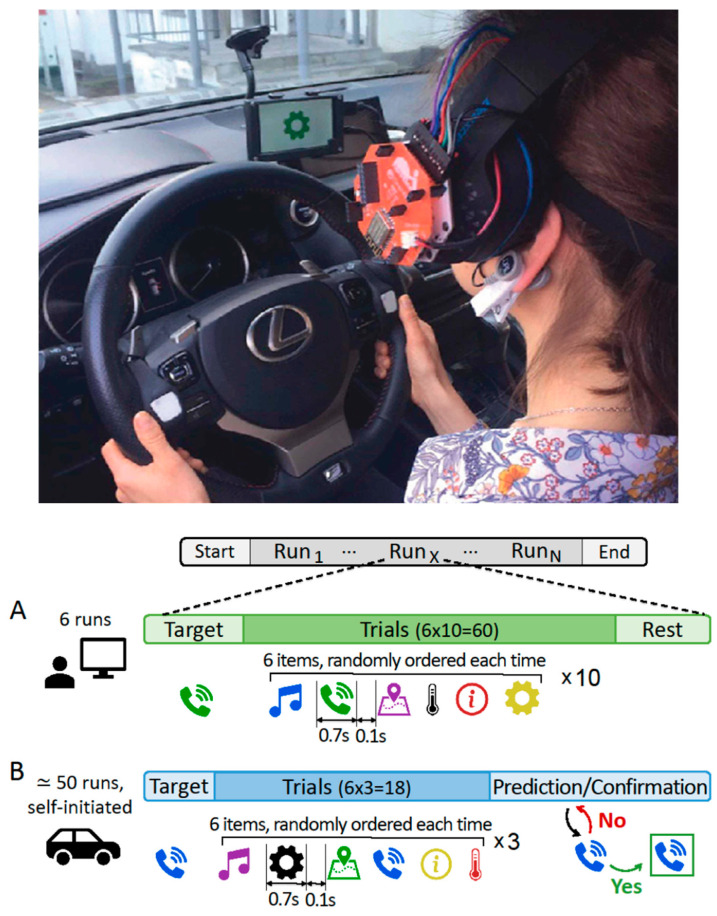
Visual representation of the BCI tasks. Upper picture shows a participant from the back wearing the EEG headset of the Biohub and performing the in-car session of the experiment. Panels (**A**,**B**) show the schema of the protocols used in the in-lab and in-car sessions.

Each participant first performed two sessions in a standard laboratory environment seated in front of a screen, and then three sessions while driving a real car in controlled conditions on a closed private proving ground (see Figure 4 for a visual representation). Sessions of each participant were performed on different days. The purpose of the initial laboratory recordings was three-fold: (i) to allow the comparison of the signal quality obtained in the two environmental conditions, (ii) to train a personalized deep learning classification model to discriminate, on a single-trial basis, between target and non-target icon appearances, and (iii) to tune the number of icon repetitions optimizing the trade-off between selection accuracy and responsiveness (i.e., the time needed to perform the selection from the activation of the menu).

During the in-car sessions, the participants were asked to drive at a constant speed and freely activate the system utilizing a button placed on the steering wheel. At the end of the carousel, the participants were provided with online feedback produced by their personalized classification model.

Participants activated the BCI-menu and provided feedback for the correctness of the BCI predictions using two buttons placed on the steering wheel (see Figure 4). The stimuli were presented on a 5-inch LCD screen attached to the windshield, driven directly by the Jetson TX2. Every session consisted of around 50 runs (composed of 18 trials as each menu icon was repeated 3 times) that were self-initiated by the participant. At the beginning of every run, a random icon was proposed to the participant as target and at the end of the carousel the ERP-based prediction was computed and presented. If the predicted icon was acknowledged as incorrect, the following most probable icon was shown, and this process continued until the correct prediction appeared.

#### 3.3.3. EEG Recordings

Only Cz, Pz, and Fp1 were considered, the latter used uniquely for rejection of artifacts due to ocular activity. Continuous EEG was acquired using the elastic OpenBCI-based EEG prototype cap at a sampling rate of 500 Hz, placing reference and ground on one ear lobe. The signals were wirelessly transmitted to the Biohub, where they were stored and processed to provide online feedback in case of driving sessions.

#### 3.3.4. EEG Analysis

The EEG data signals were band-pass filtered between 0.1 Hz and 30 Hz, and the time interval from 0 to 0.7 s after each icon appearance was epoched and baseline-corrected against the first 100 milliseconds. The epochs affected by more than 20 consecutive lost samples or abnormal amplitudes in any of the channels (greater than 100 µV) were rejected. The Biohub was evaluated in terms of signal quality and classification accuracy.

Signal quality was quantified by comparing grand average ERP characteristics such as peak amplitude and latency, PSD, and RMS obtained in the two experimental conditions and the overall percentage of rejected epochs. Additionally, to investigate the relationship between signal quality and road characteristics, we subdivided the epochs into different regions based on the GPS location of measurement and evaluated the percentage of rejected epochs in each region due to bad signal quality.

The accuracy was assessed by evaluating four classification models (Random Forest on 11 features per channel, three different deep convolutional neural networks (CNN) architectures on raw signals), and all the possible combinations of number of channels, number of icon repetitions, and acquisition location (i.e., only laboratory recordings, only in-car recordings and the two together to form a hybrid solution).

EEG data were processed using the MNE Python library and custom code. Models were built on the EEG data of the laboratory session, trained offline on a high-performance multi-GPU workstation and run online on the Biohub (Jetson module) in the in-car experiments, together with the code operating the BCI. All the computation was performed in real time by the Jetson-hosted Biohub.

## 4. Results

In this section, we report the results related to (i) the test-bench characterization of the multistream synchronization of the Biohub system, (ii) the electrophysiological validation of the EEG headset in standard laboratory conditions, and (iii) to the BCI application in a real driving scenario.

### 4.1. Characterisation of Multistream Synchronisation of the Biohub System

**Figure 5:**
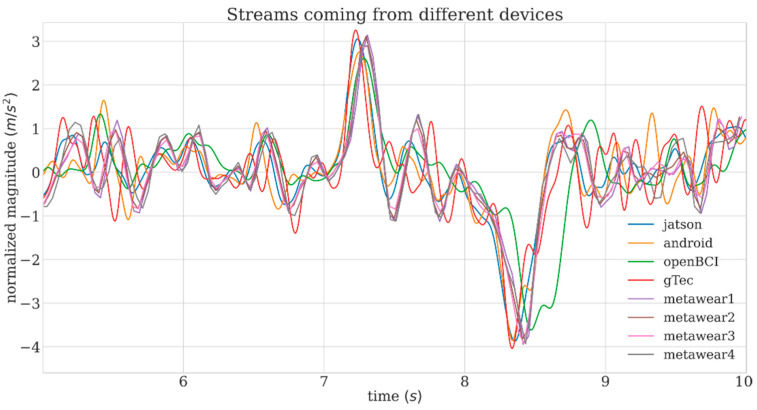
Example of accelerometer LSL-streams coming from different devices, before initial offset compensation.

Figure 5 shows a typical time course of the inertial signals from the four accelerometers hosted in the Biohub hardware nodes and the 4 external IMUs, recorded during the administration of one mechanical stimulus. From seconds 7 to 9, a continuous perturbation is visible (originating from a rigid movement of the holder).

Table 1 presents the average lags (offsets) obtained computing the cross-correlation between a reference stream (the Jetson TX2 unit in this case) and all other streams across the four sessions. The cross-correlation was computed considering only the relevant data chunks (i.e., those involving a perturbation). Results show average 2 ms offset with the smartphone, while offsets of 40 ms and 22 ms on average are recorded relative to the openBCI and the gTec devices. Latencies between 30 and 35 ms are recorded between master IMUs and external IMUs.

Measured averaged values of the offsets are also shown in terms of number of samples and are compatible with the intersample intervals at the different sampling frequencies. The very limited latency between the smartphone and the Jetson TX2 unit is expected, and it is due to the presence in both units of an LSL node and to the fact that the accelerometers are internal. The effect of the BLE transmission over external IMUs latencies is negligible given the lower sampling rates.

Conversely, 40 ms and 22 ms are the average delays of the openBCI and the gTec, respectively, and they are entirely due to the wireless transmission (WiFi and proprietary, respectively, both not managed by LSL) before reaching the repsective LSL nodes (hosted in PCs).

The large standard deviations indicate that these latencies vary at each system startup and can not be assumed constant: this was expected given the BLE and WiFi handshaking protocol. For perfect compensation, this calibration (the mechanical stimulus, estimation of initial latencies, use of this value for realignment) is to be repeated at each system startup. Runtime clock-drift estimation and dejittering are taken care for by LSL.

### 4.2. Electrophysiological Validation of the EEG Biohub Module

The Biohub system was used to acquire data from a novel EEG headset, then tested and compared to a commercial state-of-art high-density EEG acquisition system, i.e., EGI Geodesic (Electrical Geodesics Inc., Eugene, OR, USA). Overall, the electrophysiological data show that the EEG features resulting from the acquisitions with the Biohub system are comparable with those achieved with the commercial EGI Geodesic. However, the data collected with the novel system are characterized by a higher noise level, especially when considering time depending on EEG features.

**Figure 6:**
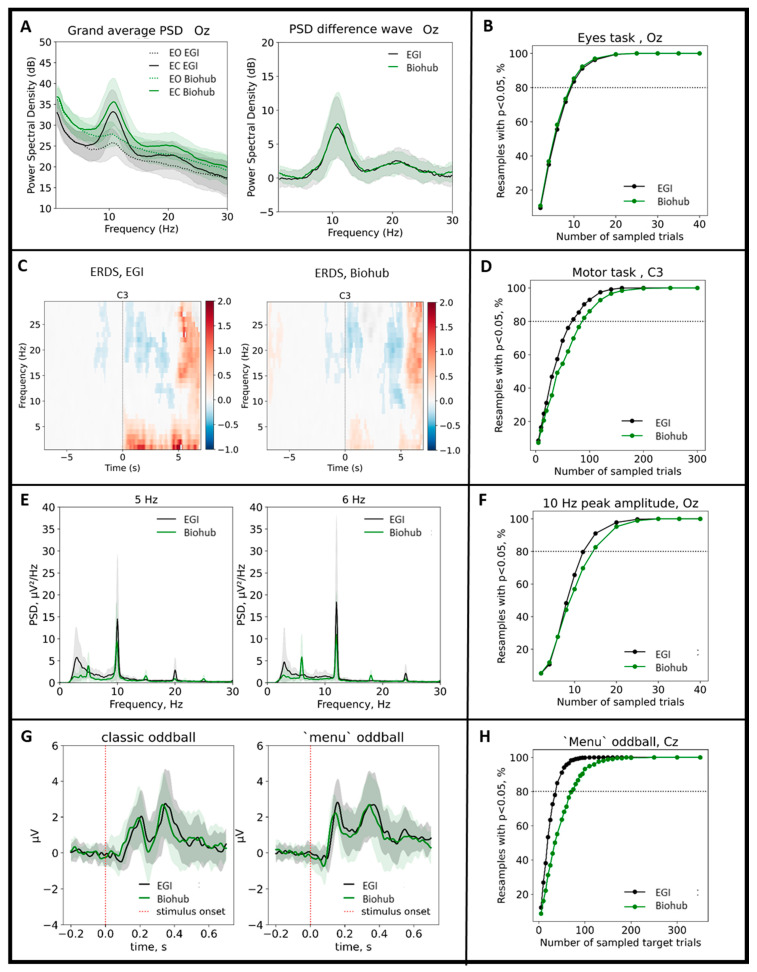
EEG features and corresponding statistical power computed for the eye (panels (**A**,**B**)), motor (panels (**C**,**D**)), SSVEP (panels (**E**,**F**)) and oddball tasks (**G**,**H**) to compare the performance between the Geodesic (black) and the Biohub (green) system. Shaded colors show standard deviations around mean.

Results are summarised in Figure 6. The picture comprises several panels illustrating the comparisons between the EEG features collected with the Biohub and the EGI systems (left side) and the corresponding analysis of statistical power across trials to measure signal-to-noise-ratio (right side). In the first row, the left panels illustrate the variation of the PSD during the eyes open-closed task for the Biohub (green) and the EGI (black) sessions for the Oz channel. The statistical comparison of the PSD amplitudes for each frequency bin in the [8, 12] Hz range indicates that there is no significant variation between the two acquisition systems for both eyes open (*t*-test, *p* > 0.05, Bonferroni corrected) and eyes closed (*t*-test, *p* > 0.05, Bonferroni corrected). Results of the motor task also returned no significant variation of the amplitude of power spectrum derived by the time-frequency decomposition of the EEG between the two acquisition systems for the C3 channel (similar results are obtained for the channels Cz and C4). Specifically, we observed a significant desynchronization of the sensorimotor rhythm during the execution of the motor task when compared to the resting period for all the recording channels covering the motor cortex. Importantly, such EEG feature is comparable between the Biohub and the EGI system (*p* > 0.05, cluster-corrected, panel C). SSVEPs collected with the Biohub, and the EGI systems returned no significant difference when the corresponding peaks of the PSD were compared for both stimulations at 5 and 6 Hz for the Oz channel (*t*-test, *p* > 0.05, panel E). Lastly, the P300 waveforms elicited by the ‘classic’ and ‘menu’ versions of the oddball paradigm were comparable between the two recording systems. Panel G shows the characteristic positive deflection around [250, 400] ms of the ERP at Cz with no significant differences between the Biohub and EGI recordings when comparing the amplitude values in the above time window of interest (*t*-test, *p* > 0.05, Bonferroni corrected).

Right panels report the proportion of the 1000 permutations exceeding the significance threshold as a function of the number of trials in each permutation. The PSD values at 10 Hz for the Oz channel measured during the eyes open-closed task with the Biohub and EGI systems exceeded significance on 80% of permutations (80% power dashed line) with equal numbers of trials (Biohub and EGI trials = 10). Similar consideration can be made for the SSVEP task, for which the significance at Oz with the 5 Hz stimulation is exceeded for a similar number of trials between the two recording systems (Biohub trials = 15, EGI trials = 11; similar results are obtained with the 6 Hz stimulation). Differently, we report that the EGI system resulted in fewer trials to exceed 80% power for the motor task calculated through the average value of ERD at C3 channel (Biohub trials = 95, EGI trials = 75; similar results were obtained with at Cz and C4 channels). In addition, the EGI system resulted in fewer trials to exceed 80% power for the oddball task, calculated through the peak of the P300 at Cz (Biohub trials = 60, EGI trials = 40; similar results are obtained at Pz). Finally, we observe that motor and P300 tasks, characterized by time-dependent features, require more trials to exceed 80% power when compared with the eyes open-closed and SSVEP tasks.

### 4.3. Validation of the Biohub EEG Module in a Car

In terms of quality of signals acquired with the Biohub in real-drive conditions, 9.3% of epochs were rejected overall, and we did not find a statistically significant difference between rejections in the straight and the corner sections, although the mean rejection rates were higher in the corners (straightaways: 8.4% ± 5.7%, corners: 10.8% ± 6.9%, *p* > 0.05).

**Figure 7:**
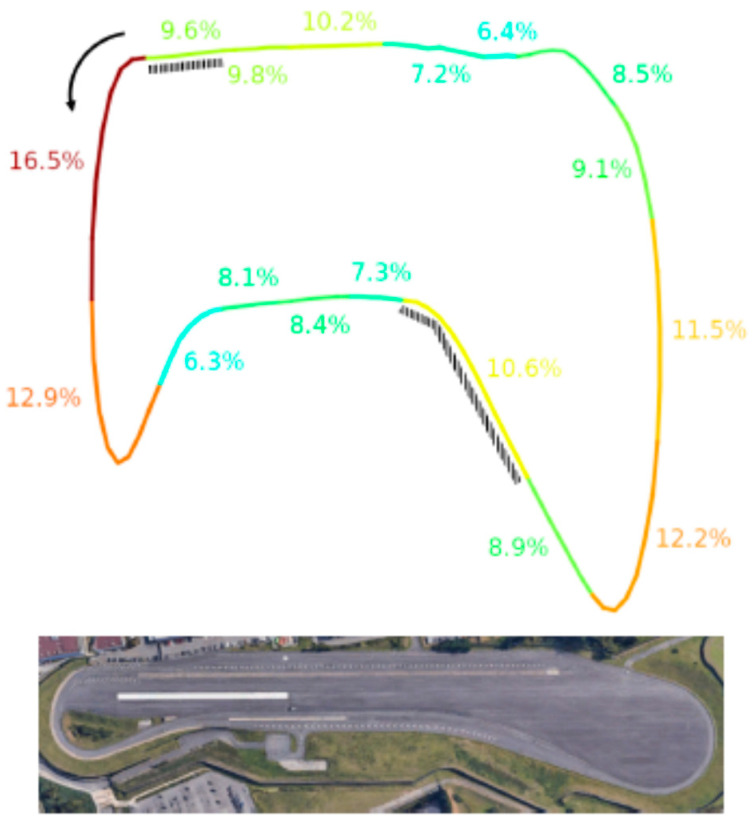
Epoch rejection rates of discarded trials in the different sections of the proving ground. The sections are color-coded based on the magnitude of the corresponding rejection rates. The arrow on the top-left corner indicates the driving direction, and the dashed markers highlight the lane-merging sections. The bottom aerial picture shows the real proportions of the proving ground.

Figure 7 shows that the greatest rejection rates were experienced in the corners and the regions immediately before them. This aspect is likely due to the merging lanes requiring the drivers to check for the presence of other cars. Nevertheless, the highest rejection rates remained as low as 17%.

In terms of classification accuracy, multiple configurations were evaluated to identify the best classifier to the EEG P300 responses as target or non-target.

Firstly, three different training dataset configurations were investigated: only laboratory data, only car data, and a hybrid obtained combining the former two. The latter configuration provided the best results on the separate test set consisting of a separate in-car session.

**Figure 8:**
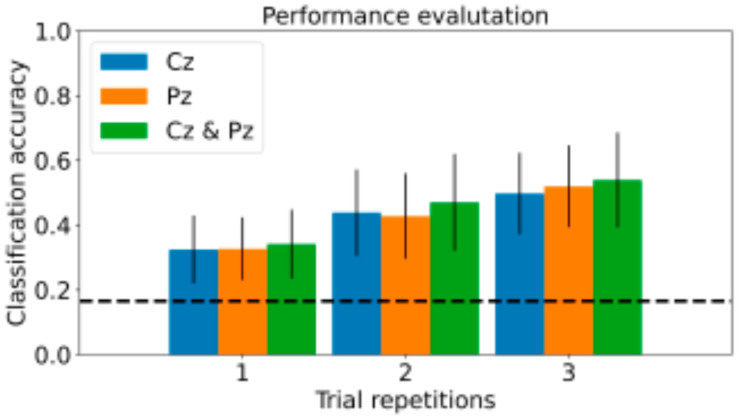
Average subject-specific classification accuracy varying EEG channels configuration and the number of repetitions of the menu icons.

Secondly, the impact of number of repetitions and electrodes configuration on classification performance was investigated. Figure 8 shows all the evaluated combinations with the corresponding average classification accuracy among the 10 participants. As expected, we observed a better performance when increasing the number of repetitions and adopting more channels (54% accuracy against 16% of the random-choice level).

Overall, the combination of 3 channels and 3 repetitions proved to be a viable trade-off between comfort, system response time, and performance. In addition to the subject-specific models, we trained a single general model of the same CNN architecture by grouping the data of the 10 participants. The performance on the separate in-car test set, consisting of the combination of the original 10 test sets, remained three times higher than random chance (52%), confirming the identification of common patterns across multiple participants despite the complex dual-task (icon selection while driving) and the narrow area covered by the minimal adopted montage.

The general model was then evaluated in the laboratory condition on five new participants, whose signals were not considered in the training phase, and the performance remained as high as 49% ± 17%. Despite preliminary, this finding supports the generalisability of the results achieved with the described BCI.

## 5. Discussion

In the present study, we introduced the Biohub platform, a device capable of measuring human brain dynamics in applied environments. We provided (1) a description of the hardware/software architecture, (2) validation of its synchronization capacities in multimodal acquisitions, (3) validation of the EEG signal acquired through Biohub against a medical-grade high-density EGI device, and finally, (4) a demonstration of the Biohub applicability in a real-life ecological scenario such as car driving. Overall, this evidence supports the usability of Biohub in the field of neuroergonomics, being a flexible platform able to reliably acquire, accurately synchronize and record multiple streams of various signals related to the human brain and behavior in everyday life settings [14,34,35].

Biohub is a modular architecture of networked nodes, which can be hosted on personal computers and smartphones or embedded systems such as the Jetson TX2 system-on-board from NVidia for maximal portability in out-of-lab situations. An orchestrator node allows launching and monitoring nodes, operating at potentially different sampling frequencies; a specific multimodal GUI allows online and offline replay of all streams. The LSL middleware ensures the synchronization across streams wherever a TCP/IP connection can be established. We developed two plugins to overcome the potential issues related to LSL compatibility and the limited computational power of the several commercial devices interfaceable with Biohub. The first enables LSL streams from Android smartphones receiving data from Bluetooth sources not compatible with the TCP/IP protocol. The second plugin allows LSL-streaming of TCP/IP sources too computationally limited to host an LSL client. Both plugins implement techniques to maximize the synchronization across non-LSL-assisted wireless streams over the whole duration of the acquisition. The efficiency of these solutions for a proper temporal synchronization among data streams is guaranteed by a calibration procedure, which is worth being conducted before the acquisitions, thus allowing accurate, LSL-assisted, online inter-stream adjustment. The Biohub system acquires the ideal set of signals related to the brain and behavioral dynamics, allowing the development of real-life applications based on human-machine interaction. The overall easiness of application of the Biohub is an adjunct value, especially in experimental settings with infants, the elderly, professionals, and patients in the hospital. Taken together with the high temporal stability of Biohub across successive recording sessions, these aspects are fundamental for high-throughput applications in which multimodal recordings might be crucial for action monitoring and prediction in critical tasks such as driving [16,19,33], neurorehabilitation [36,37] as well as other strategic scenarios [38]. In comparison with existing commercial devices, the Biohub custom hardware and software architecture eases the possibility (and lowers the costs) to couple different physiological, behavioural, and contextual measures for online data analyses in real-life applications. The highly programmable interface enables the design of a broad set of studies in cognitive neuroscience using, for instance, contextual measures to trigger an adaptive stimuli presentation. The overall Biohub architecture, including its inter-stream synchronization, was “stress-tested” in the in-car BCI paradigm, which required the online application of advanced machine learning models for time series analysis. The successful findings reported in this test-case indicate the Biohub as a hybrid systemenabling the setting up of paradigms for measuring different psychophysiological variables, with better performance relative to conventional, unimodal BCI systems [20,39].

To test the quality of the EEG signals recorded by Biohub, we compared in the same participants the scalp signals recorded during four experimental tasks with those collected by a certified, medical-grade lab device. These tasks were proposed to elicit neural features traditionally exploited in BCI settings. In the frequency domain, we measured the reactivity of the occipital visual rhythms (during the eyes-open/closed task), the reactivity of central motor rhythms (during a motor execution/rest task), and the amplitude of the steady-state visually evoked potentials. In contrast, in the time domain, we investigated the modulation of the P300 potential during a six-class oddball task. We observed that the neural signatures collected with the Biohub platform parallel those collected with the EGI system, as reflected in the lack of differences across all four experimental tasks. Evidence that traditional EEG measures can be replicated with a dry electrode system is promising.

According to a resampling procedure estimating the number of trials necessary to achieve a certain level of statistical power [40], the recordings performed with the Biohub system are characterized by a higher signal-to-noise ratio relative to the EGI system for motor and oddball tasks, but not for the visual ones. In turn, this requires a larger number of trials for Biohub relative to the EGI system to achieve comparable levels of power. This aspect might, in principle, limit the benefits in terms of convenience and flexibility of the Biohub. Several aspects, however, must be taken into account since the initial experimental design. First, dry electrodes are characterized by higher impedance than passive, active, and wet sensors, and dry systems are more sensitive to temporal than spectral neural features [41,42,43,44].

Moreover, even the topography of the sought neural features affects the capacity of dry systems to match the signal-to-noise ratio achieved via wet systems. Indeed, our findings indicate the high suitability of Biohub for measuring occipital, visual signals with a quality comparable to the EGI system, whereas poorer performance is obtained when dealing with more frontal features. In summary, a proper trade-off must be established for each application, first identifying the targeted neural features and then balancing the desired quality of the recorded signal (wet are superior to dry) with the easiness of application (dry are almost 10 times faster than wet systems).

The Biohub system was also validated within an ecologic driving-related BCI scenario task, which consisted of a car driver on the track operating on an infotainment menu (as a secondary task) via the EEG signal, with the selection mechanism based on P300 potential. Of note, the classification accuracy reached by drivers operating the infotainment was 52%, which is three times above the chance level. Beyond the overall and successful accuracy, the multistream synchronization proved essential to precisely correlate the discarded chunks of data (due to the presence of excessive artifacts) with the position along the circuit, revealing, for instance, that the discard rate was higher for a specific turn requiring wider head movements.

Beyond driving, the Biohub system could foster the spread of BCI applications onto several other contexts of our daily life. The possibility to remotely monitor an individual’s brain and mental state would have a tremendous impact on telemedicine applications, particularly those related to the rehabilitation field [45], with the possibility to verify the compliance and adherence to treatment relieving the patient and caregivers from a massive burden in terms of time and costs. Applications could also extend beyond the clinical realm, virtually to any fields where surveillance of the brain and body interplay would be valuable for preventing harmful consequences. It is the case, for instance, of the occupational safety of workers dealing in their routine with unsafe practices, for whom the use of a platform such as Biohub could reduce the likelihood of occupational injuries during the performance of high-risk motor tasks [46], as well as signal the appearance of negative mental states such as excessive workload and fatigue, or reduced alertness and vigilance [21].

Numerous public and private actors are envisioning the deployment of BCI in novel clinical and industrial settings in the near future [47]. Some programs, such as the BNCI Horizon 2020 project [48], have established a future roadmap for BCI systems. Nevertheless, the BCI community should address and consider critical limitations, challenges, and issues related to BCI paradigms and platforms [11]. However, with the growing increase of EEG-based BCI studies concerning the development of wearable amplifiers, sensors, algorithms, and psychophysiological aspects of the EEG signal, we believe that the large-scale deployment of further BCI applications is a matter of years.

## Figures and Tables

**Table 1:** Computed offsets (in ms) between streams (the first row contains those relative to the master IMU, i.e., the Jetson TX2 in this case) averaged across sessions. The number in [] indicates the offset in terms of samples given the different sampling rates.

	Jetson	Android	gTec	openBCI	Metawear1	Metawear2	Metawear3	Metawear4
jetson	0 ± 0	−2 ± 8 [1]	−22 ± 12 [11]	40 ± 2 [20]	−35 ± 11 [1]	−31 ± 7 [1]	−30 ± 8 [1]	−34 ± 10 [1]
android		0 ± 0	−21 ± 5	33 ± 11	−30 ± 22	−29 ± 17	−24 ± 20	−32 ± 22
gTec			0 ± 0	63 ± 1	−23 ± 31	−14 ± 20	−9 ± 19	−16 ± 22
openBCI				0 ± 0	−68 ± 8	−63 ± 9	−64 ± 6	−71 ± 13
metawear1					0 ± 0	3 ± 3	4 ± 4	−1 ± 1
metawear2						0 ± 0	0 ± 0	−4 ± 2
metawear3							0 ± 0	−4 ± 2
metawear4								0 ± 0

## Data Availability

The data that support the findings of this study are available from CAMLIN Limited but restrictions apply to the availability of these data, which were used under license for the current study, and so are not publicly available. Data are however available from the authors upon reasonable request and with permission of CAMLIN Limited.
